# Hints to Pew Owners

**Published:** 1856-08

**Authors:** 


### HINTS TO PEW OWNERS.

In entering city Churches, persons are often seen standing, waiting for the sexton to show them a seat; if they have to wait long, the temper of many becomes ruffled and they either leave the house or take their seats in a state of mind unsuited to the solemnities of religious service. We propose our own practice, as a means of lessening the evil. We know before leaving home how many seats will be occupied by the members of our family, and in case of a known vacancy, we conduct any stranger at the vestibule to our own pew. The very act saves time to the sexton, relieves him of the necessity of asking your permission to receive a stranger, prevents your having to get up, march out into the aisle, bow the stranger in and you following, thus discomposing your own reflections, and attracting the attention of the congregation; and above all, it excites a feeling of kindness towards you, in the stranger's bosom, as creditable and profitable to himself, as the courtesy which originated it, was to you.

